# Repurposing Screen Identifies Unconventional Drugs With Activity Against Multidrug Resistant *Acinetobacter baumannii*

**DOI:** 10.3389/fcimb.2018.00438

**Published:** 2019-01-04

**Authors:** Yu-Shan Cheng, Wei Sun, Miao Xu, Min Shen, Mozna Khraiwesh, Richard J. Sciotti, Wei Zheng

**Affiliations:** ^1^National Center for Advancing Translational Sciences, National Institutes of Health, Bethesda, MD, United States; ^2^Experimental Therapeutics Branch, Walter Reed Army Institute of Research, Silver Spring, MD, United States

**Keywords:** *Acinetobacter baumannii*, multidrug resistance, drug repositioning, drug repurposing screen, synergistic drug combination, nosocomial infections, non-antimicrobial drugs

## Abstract

Antibiotic-resistant nosocomial infections are an emerging public health issue; carbapenem-resistant gram-negative bacteria such as *Acinetobacter baumannii* are among the pathogens against which new therapeutic agents are desperately needed. Drug repurposing has recently emerged as an alternative approach to rapidly identifying effective drugs and drug combinations to combat drug resistant bacteria. We performed a drug repurposing screen against a highly virulent, multidrug resistant, *Acinetobacter baumannii* strain AB5075. This strain, isolated from a patient, is resistant to 25 first-line antibiotics for gram-negative bacteria. A compound screen using a bacterial growth assay led to identification and confirmation of 43 active compounds. Among these confirmed compounds, seven are approved drugs or pharmacologically active compounds for non-antimicrobial indications. Three of these drugs, 5-fluorouracil, fluspirilene, and Bay 11-7082 resensitized strain AB5075 to azithromycin and colistin in a two-drug combination format. The approach using a drug repurposing screen with a pathogen sample isolated from a patient and a high throughput bacterial growth assay led to the successful identification of new drug combinations to overcome a multidrug resistant bacterial infection.

## Introduction

The emergence and dissemination of drug-resistant bacterial infections are a public health issue. *Acinetobacter baumannii* is one of the major causes for the nosocomial infections in critically ill patients. Treatment of *Acinetobacter baumannii* can be extremely difficult, especially for the carbapenem resistant strains. Colistin and tigecycline are the last resorts for carbapenem resistant *Acinetobacter baumannii*. However, colistin and tigecycline resistant strains have been reported worldwide (Deng et al., 2014; Oikonomou et al., 2015). In light of the rapid expansion of imipenem resistance in 35 countries, increasing from 24 to 74% in just 11 years (Xie et al., 2018), the development of novel drugs to combat *Acinetobacter baumannii* infections is an urgent need.

Antibiotic development mainly relies on two strategies, a target-based approach and isolation of bioactive secondary metabolites from microorganisms (Demain, 1999; Marinelli, 2009). New antibiotic drug development is a long-term process; for example, a target-based method takes time to go through the steps of target selection, lead discovery and optimization, preclinical development, then clinical trials before the FDA gives approval for marketing for a new indication. Drug repurposing and drug combinations have emerged as promising alternative approaches to provide novel therapeutic options for multidrug resistant bacteria (Zheng et al., 2018). Drug repurposing of approved drugs bypasses the need for novel molecules in nature or from a synthetic chemical library, and alleviates the need for preclinical development and phase I clinical trials as the data for pre-clinical experiments, human pharmacokinetics, and drug safety are already established. Thus, drug repurposing accelerates the drug development process and reduces the development costs. In addition, drug combination therapy with a synergistic effect of two or three drugs in combination can overcome drug resistance by inhibiting multiple targets and reducing occurrence of further drug resistance.

A multidrug resistant *Acinetobacter baumannii* clinical isolate, *Acinetobacter baumannii* 5075 (AB5075) (Jacobs et al., 2014), was used in this study. Strain AB5075 was first isolated from the osteomyelitis of a patient's tibia bone in 2008. The detailed genomic analysis of AB5075 in 2015 unveiled some antibiotic resistant mechanisms (Gallagher et al., 2015). Briefly, AB5075 carries 133 genes that likely cause resistance to broad-spectrum β-lactams (for example, penicillins, cephalosporins, and carbapenems), aminoglycosides, chloramphenicol, quinolones, tetracycline, trimethoprim, sulfonamides, macrolides, and other toxic agents. We report here the identification of seven non-antimicrobial drugs that suppressed AB5075 growth *in vitro* by a drug repurposing screen using the AB5075 strain. The results demonstrate the usefulness of a drug repurposing screen using patient derived pathogens. These newly identified compounds with inhibitory activities against multidrug resistant *Acinetobacter baumannii* can be further studied for use as new therapeutic agents.

## Materials and Methods

### Materials

Tigecycline was obtained from Chem-Impex International (Wood Dale, IL, USA). Doripenem and ertapenem were acquired from Cayman Chemical (Ann Arbor, MI, USA) and TOKU-E (Bellingham, WA, USA), respectively. Other chemicals were purchased from Sigma-Aldrich (St. Louis, MO, USA).

### Preparation of Bacterial Stock for High Throughput Screen

*Acinetobacter baumannii* 5075 (AB5075) was obtained from Walter Reed Army Institute of Research. Individual colonies on agar plates were cultured in tryptic soy broth (TSB, Remel, Thermo Scientific, Waltham, MA, USA) at 37°C. The bacterial cultures were mixed with sterile glycerol in a 9:1 ratio when the optical density at 600 nm (OD_600_) reached about 0.25–0.3. Bacteria in 10% glycerol were stored in aliquots at −80°C.

### Bacterial Growth Experiments

Bacteria were thawed from −80°C and diluted to a desire initial density from 1:200 to 1:1,000 in TSB. Each bacterial culture was grown in TSB at 37°C, 5% CO_2_ humidified atmosphere for 2–48 h. Bacterial growth was monitored by measuring the OD_600_ in a PHERAstar plate reader (BMG Labtech, Cary, NC, USA).

### Compound Library

A pharmacologically active compound library (LOPAC 1280) was purchased from Sigma-Aldrich (St. Louis, MO, USA). The NCATS Pharmaceutical Collection (NPC) of approved and investigational drug collection was generated in house (Huang et al., 2011). The NPC library consists of 2,816 small molecule compounds, 38.4% approved by U.S. FDA, 22.5% approved in the EU, Canada, or Japan, and 39.0% being used in clinical trials or as research compounds.

### Compound Screening and Validation

A quantitative high throughput screen (qHTS) and confirmation assays were performed as previously described in a 1,536-well format (Sun et al., 2016). Briefly, 2.5 μL TSB was first loaded into each well of a black clear bottom microplate by a Multidrop Combi dispenser (Thermo Fisher Scientific, Waltham, MA, USA). An automated pintool station (WAKO Scientific Solutions, San Diego, CA) was then used to transfer 23 nL of compounds from compound plates into assay plates. For the primary screen, each compound was tested at four concentrations. Compound plates were prepared by an Evolution P^3^ system (PerkinElmer, Wellesley, MA) as described in a previous publication (Inglese et al., 2006). In the follow-up confirmation assays, dose-effects of the carefully selected compounds were examined as an intraplate 11-point, 3-fold dilution series. Each compound was tested in three biological replicates. Dimethyl sulfoxide (DMSO) served as a negative control and 46 μM of rifampin was the positive control. Bacterial stock diluted in TSB at a ratio 1:500 was added at 2.5 μL/well to make a 1:1000 dilution of initial inoculum density. The assay plates were incubated at 37°C for 20–22 h and placed onto a PHERAstar plate reader (BMG Labtech, Cary, NC, USA) to detect the bacterial growth in response to OD_600_.

### Two Drug Combination Assays

In two drug combination assays, non-antimicrobial drugs (drug 1) resulting from the qHTS were tested in combination with an 11-concentration series of 25 standard treatment drugs (drug 2). The screen was conducted in the same procedure as for qHTS except the TSB media was mixed with drug 1 prior to loading into the assay plates. The final concentrations of drug 1 were one-fourth and one-eighth of the calculated half-maximal inhibitory concentration (IC_50_) values. The dose-effect curve of drug 2 as a single agent or with a fixed concentration of drug 1 were calculated. A significant synergistic response was defined as a three-fold decrease in IC_50_. Drug pairs that showed a synergistic response were further validated in a 96-well plate.

### Statistics and Data Analysis

The qHTS analysis was designed internally to include three steps: normalization, pattern correction, and curve fitting. Raw plate reads were first normalized to relative controls (such that DMSO alone was considered as 100% viability and 46 μM rifampin as 0% viability) using the following equation: % normalized viability = [(V_compound_ –V_positive_)/(V_DMSO_ –V_positive_)] × 100. Next the data underwent a pattern-correction based on information from a DMSO-alone plate. Interplate dose-response data of each compound was processed by a four-parameter Hill equation based grid algorithm to yield the IC_50_, the maximum response, and the curve class (Wang et al., 2010). IC_50_ and IC_90_ values in the confirmation experiments were calculated with Prism 7 software (GraphPad Software, Inc. San Diego, CA, USA).

## Results

### Assay Optimization for the High Throughput Bacterial Growth Assay

**Figure 1:**
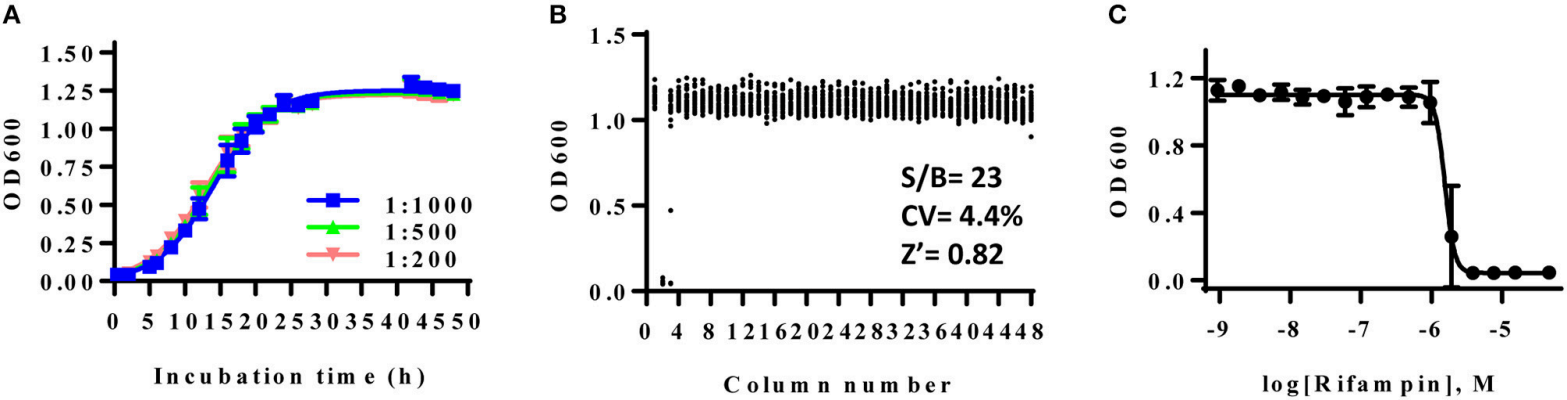
**(A)** Growth curve of AB5075 in 1536-well plate. AB5075 stock solution was diluted to different starting ratios and incubated at 37°C. Data points represent the mean, and the error bars represent the standard deviation (SD); *n* = 128. **(B)** Scatter plot of the results from a DMSO plate screening. The wells in column 2 of the 1536-well assay plate contained 46 μM rifampin as a positive control (0% viability); the wells in column 3 contained varying doses of rifampin at 1:3 dilution. The wells in the rest of plate contained DMSO as a negative control (100% viability). The signal-to-basal ratio (S/B) in this plate was 23-fold, with a coefficient of variation (CV) of 4.4%, and a Z′ factor of 0.82. **(C)** Dose–response curves for rifampin from column 3. The data points represent the mean, and the error bars represent the SD; *n* = 2.

The high throughput bacterial growth assay (Sun et al., 2016) were adapted to examine antibiotic susceptibility for AB5075. The growth kinetics of AB5075 was monitored in a 1536-well plate format to determine the optimal inoculum density and incubation time. Measurement of OD_600_ of assay plates, reflecting the bacterial growth rate, was recorded at various time points over 48 h (Figure 1A). A classical growth kinetic pattern was observed encompassing lag, exponential, and stationary phases; the growth reached stationary phase at 24 h. As suggested by the antimicrobial susceptibility testing guideline for *Acinetobacter* spp. from the Clinical and Laboratory Standards Institute (CLSI), experimental endpoints were setting at 22–24 h for the following studies. All bacterial stock dilutions used for inoculum showed similar signal-to-basal ratios of ~26-fold. The dilution of 1:1,000 was chosen for use in further experiments.

The DMSO alone plate was used to account for well to well variation. Rifampin was selected to serve as the positive control compound in the experiments. The calculated signal-to-basal ratio was 23-fold, the coefficient of variation (CV) was 4.4%, and the Z′ factor was 0.82, indicating a robust assay for high throughput screening (Figure 1B). The calculated IC_50_ value of rifampin against AB5075 was 0.40 μM (Figure 1C).

### Activities of Standard Care Antibiotics Against AB5075 Strain

**Figure 2:**
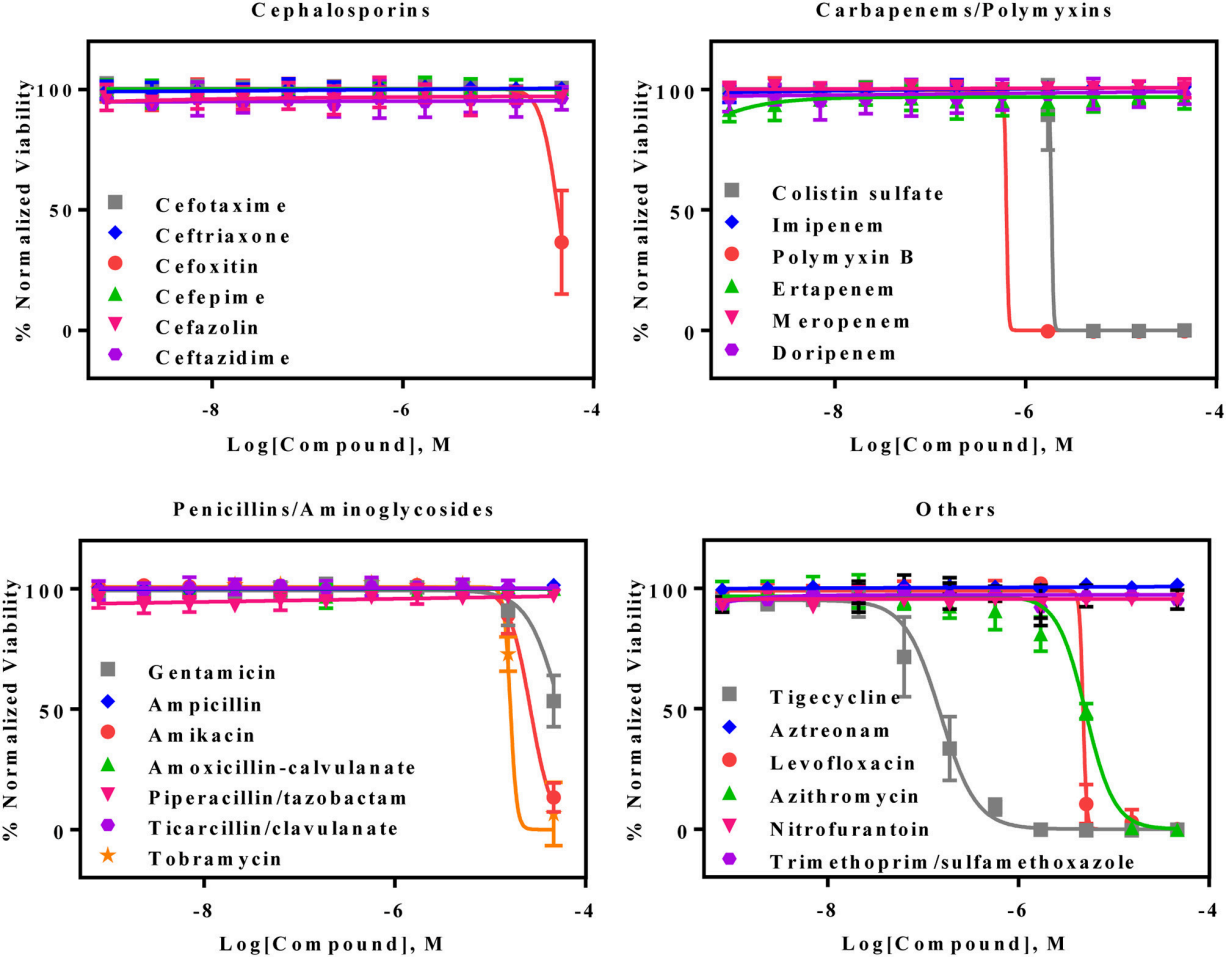
Concentration–response curves of the inhibition of AB5075 growth by 25 standard antibiotics. Data points represent mean, and error bars represent the SD; *n* = 3.

The AB5075 growth assay was evaluated with a set of 25 antibiotics commonly used for infections by gram negative bacteria. Nine compounds showed concentration-dependent inhibition of AB5075 growth including tigecycline, two polymyxins (polymyxin B and colistin sulfate), levofloxacin, azithromycin, three aminoglycosides (amikacin, gentamicin, and tobramycin) and cefoxitin (Figure 2, Table 1). Among these active antibiotics, tigecycline was the most potent compound with an IC_50_ of 0.15 μM. The other 16 antibiotics were not active (i.e., IC_50_ > 46 μM) against the multidrug resistant AB5075 strain.

**Table 1:** IC_50_ and MIC data for standard care antibiotics against AB5075.

**Antibiotics**	**IC_**50**_**	**IC_**90**_**	**Reported MIC**		**Breakpoints for resistance**	
**Unit**	**μM**	**μM**	**μg/ml**	**μM**	**μg/ml**	**μM**
**Carbapenems**						
Doripenem	>46	>46			2	4.8
Imipenem	>46	>46	8	25.2	2	6.3
Ertapenem	>46	>46			N/A	
Meropenem	>46	>46			2	4.6
**Penicillins**						
Amoxicillin-clavulanate	>46	>46	≥32	≥87.6	N/A	
Ampicillin	>46	>46	≥32	≥86.2	16	43.1
Ticarcillin-clavulanate	>46	>46			16/2	41.6
Piperacillin-tazobactam	>46	>46	≥128	≥247.3	16/4	30.9
**Cephalosporins**						
Cefazolin	>46	>46	≥64	≥140.8	N/A	
Cefepime	>46	>46	≥64	≥112.0	8	14.0
Cefotaxime	>46	>46	≥64	≥134.1	8	16.8
Cefoxitin	42.0	66.5	≥64	≥142.4	N/A	
Ceftazidime	>46	>46	≥64	≥100.5	8	12.6
Ceftriaxone	>46	>46	≥64	≥96.7	8	12.1
**Aminoglycosides**						
Amikacin	26.3	46.8			16	20.5
Gentamicin	54.8	>46	≥16	≥10.8	4	2.7
Tobramycin	16.3	39.4	2	4.3	4	8.6
**Polymyxins**						
Polymyxin B	0.63	0.69			2	1.4
Colistin sulfate	1.9	2.0			2	1.6
**Macrolide**						
Azithromycin	5.1	10.8				2.5
**Quinolones**						
Levofloxacin	4.7	5.2	4–8	11.1–22.1	2	5.4
**Folate Pathway Inhibitors**						
Trimethoprim-sulfamethoxazole	>46	>46	≥320	≥1102.2	2/38	6.9
**Glycylcycline**						
Tigecycline	0.15	0.39	≤0.5	≤0.85	N/A	
**Other**						
Aztreonam	>46	>46	≥64	≥147.0	N/A	
Nitrofurantoin	>46	>46	≥512	≥2149.8	N/A	

### Repurposing Screen Using LOPAC and NPC Library

**Figure 3:**
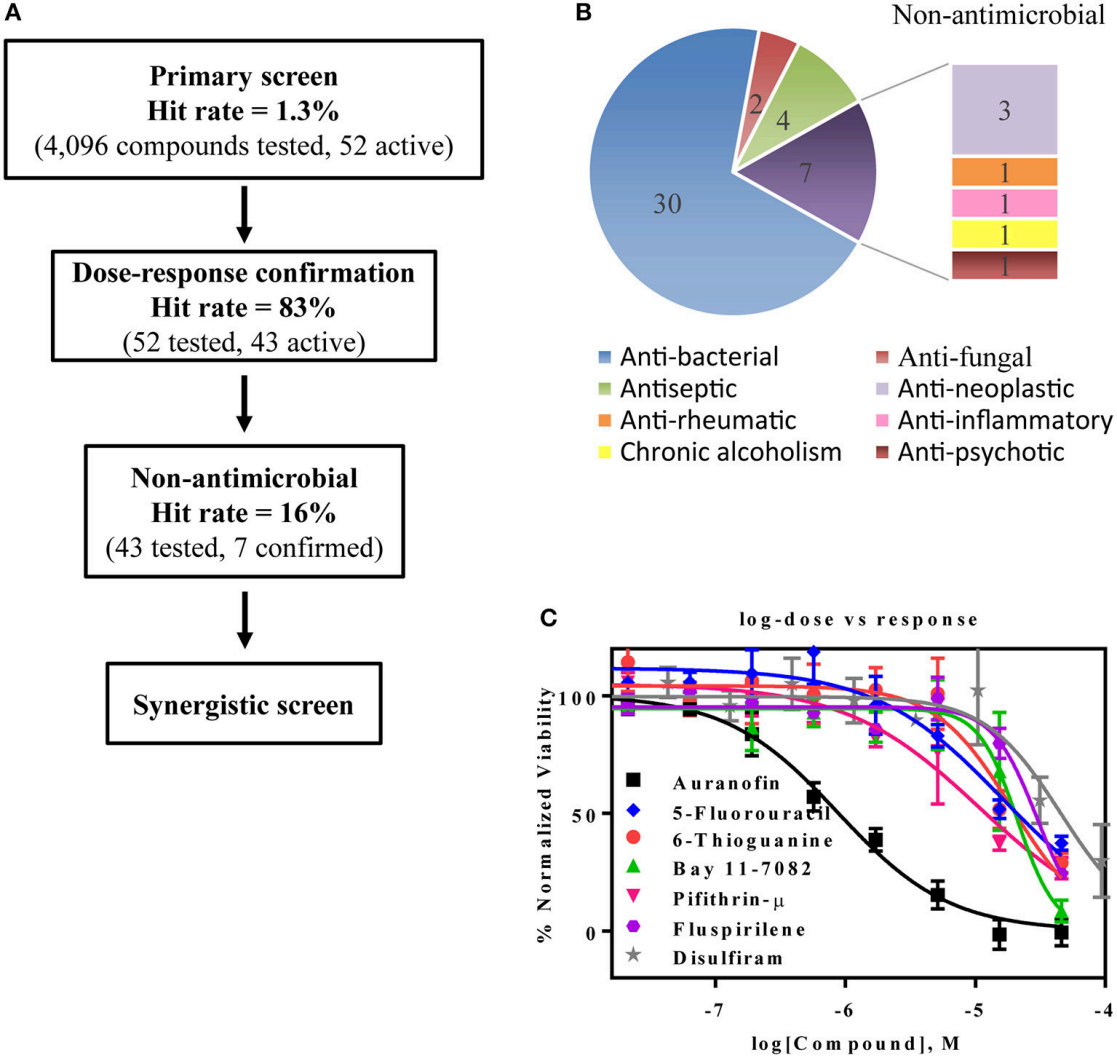
Experimental design and results of the repurposing screen. **(A)** Flow chart of the compound screening and confirmation. **(B)** Pie chart illustrates the identified compound categories. **(C)** Concentration-response curves of the non-antimicrobial compounds.

The primary screen assessed 4,096 approved drugs and bioactive compounds to identify compounds inhibiting the growth of AB5075 (Figure 3A). Each compound was tested at four concentrations (4.1, 9.2, 20.6, and 46 μM) in the primary screen. The primary hits were selected based on an IC_50_ below 30 μM and an efficacy (maximum inhibition) >70%. Fifty-two compounds meeting these criteria were selected and retested, resulting in 43 confirmed compounds, an 83% confirmation rate (Table 2). These confirmed compounds include 30 antibacterial, 2 antifungal, 4 antiseptic, 3 antineoplastic, and 4 other agents (Figure 3B). Tetracycline and its analogs were the most potent compounds identified with IC_50_ values of 0.045–0.47 μM. Among the confirmed compounds, seven were drugs categorized as non-antimicrobial agents (Figure 3C, Table 3).

**Table 2:** Antagonists identified in the qHTS.

**Drug name**	**IC_**50**_ (μM)**	**Maximum response**	**Primary action**
Doxycycline HCl	0.045	109	Antibacterial
Minocycline HCl	0.094	112	Antibacterial
Demeclocycline HCl	0.13	94	Antibacterial
Methacycline HCl	0.13	84	Antibacterial
Sancycline	0.14	104	Antibacterial
Rifampicin	0.45	100	Antibacterial
Tetracycline HCl	0.47	91	Antibacterial
Thimerosal	0.53	92	Antiseptic and germicides
Triclosan	0.93	90	Antibacterial
Auranofin	1.09	106	Antirheumatic
Gatifloxacin	1.37	105	Antibacterial
Sitafloxacin	1.43	97	Antibacterial
Novobiocin sodium	1.53	107	Antibacterial
Diphenyleneiodonium chloride	1.97	101	Antibacterial
Phenylmercuric acetate	2.77	99	Antifungal in agriculture
Sparfloxacin	3.64	106	Antibacterial
Trovafloxacin mesylate	4.18	102	Antibacterial
Erythromycin propionate	4.35	110	Antibacterial
Enrofloxacin	4.88	99	Antibacterial
Malachite green oxalate	5.07	101	Antiseptic in veterinary
Marbofloxacin	5.54	94	Antibacterial
Nitroxoline	5.79	108	Antibacterial
Nitromersol	5.90	94	Antiseptic and disinfectant.
Pifithrin-mu	7.24	82	Antineoplastic (p53 inhibitor)
Chloroxine	7.29	96	Antibacterial
5-Fluorouracil	7.29	79	Antineoplastic
Grepafloxacin HCl	8.03	96	Antibacterial
Ticlatone	8.45	88	Antifungal
Azithromycin dihydrate	8.71	117	Antibacterial
Moxifloxacin HCl	9.35	105	Antibacterial
Garenoxacin mesylate hydrate	9.38	96	Antibacterial
Ofloxacin	9.66	100	Antibacterial
Difloxacin HCl	11.34	117	Antibacterial
Fusidic acid sodium	11.34	124	Antibacterial
6-Thioguanine	11.98	77	Antineoplastic
Tosufloxacin toluenesulfonic acid	14.54	76	Antibacterial
Dipyrithione	16.86	111	Fungicidal and bactericidal
Nadifloxacin	17.30	95	Antibacterial
Alatrofloxacin mesylate	18.06	103	Antibacterial
Fluspirilene	22.74	77	Antipsychotic
Bay 11-7082	23.57	99	Anti-inflammatory
Alexidine dihydrochloride	29.02	111	Antibacterial
Disulfiram	30.08	81	Chronic alcoholism

**Table 3:** Active and plasma concentration for non-antimicrobial indication candidates.

**Drug name**	**Chemical structure**	**IC_**50**_ (μM)**	**IC_**90**_ (μM)**	**Max response**	**Primary action**	**C**_****max****_	
						**μg/mL**	**μM**
Auranofin	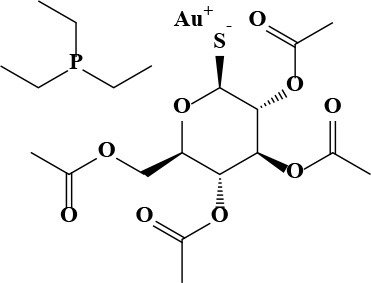	1.09	7.6	106	Antirheumatic	0.68^*^	1
Pifithrin-μ	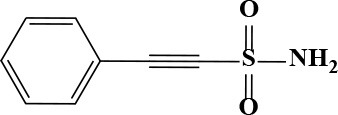	7.24	145.8	82	Antineoplastic (p53 inhibitor)	N/A	
5-Fluorouracil	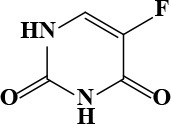	7.29	193.9	79	Antineoplastic	48.41 Bocci et al., 2000	372.2
6-Thioguanine	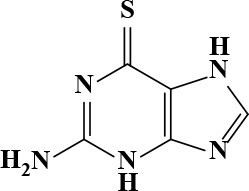	11.98	84.8	77	Antineoplastic	15 Kovach et al., 1986	87
Fluspirilene	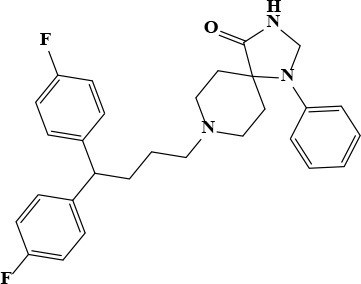	22.74	71.7	77	Antipsychotic	0.2 × 10^−3^ Swart et al., 1998	0.42 × 10^−3^
Bay 11-7082	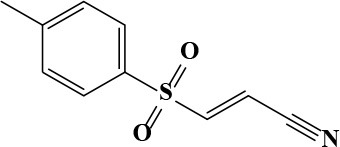	23.57	45.0	99	Anti-inflammatory	N/A	
Disulfiram	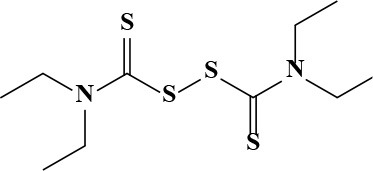	30.08	198.7	81	Chronic alcoholism	0.39 × 10^−3^	1.3 × 10^−3^ Johansson, 1992

* *http://www.prometheuslabs.com/Resources/PI/Ridaura.pdf*.

### Synergistic Drug Combinations of Newly Identified Non-antimicrobial Compounds and Standard Care Antibiotics

**Figure 4:**
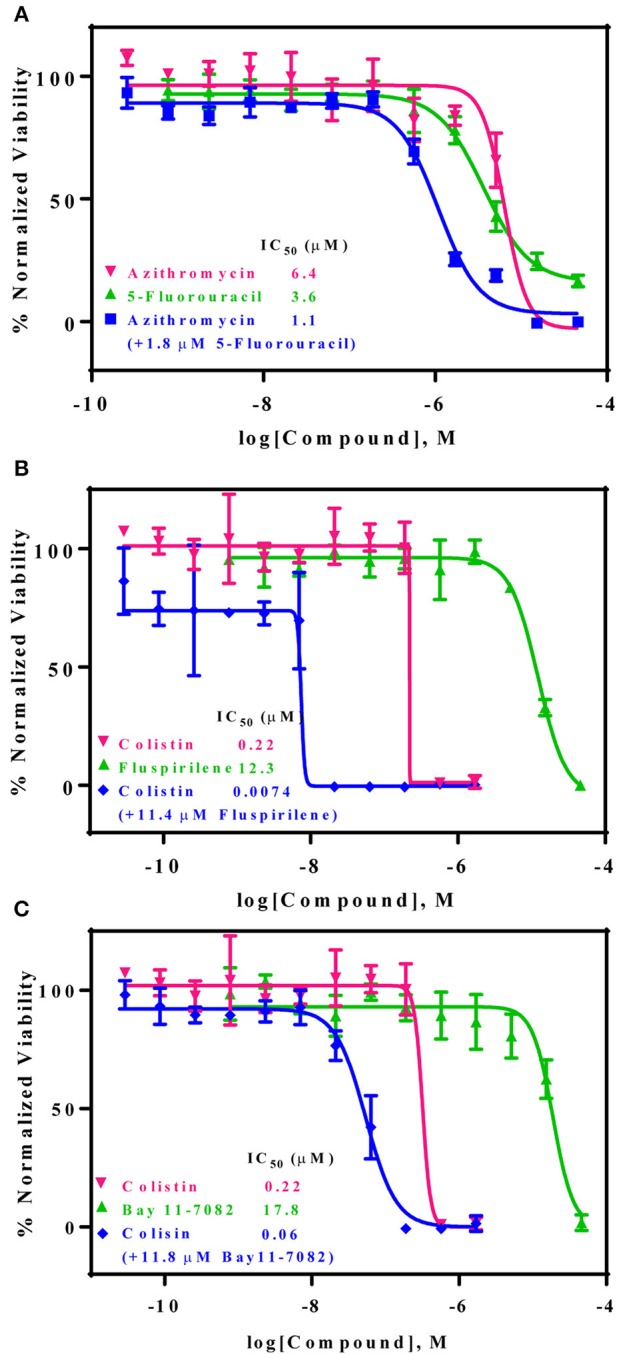
Three non-conventional active compounds resensitize AB5075 to standard care antibiotics. AB5075 was treated with 5-fluorouracil, fluspirilene, or Bay 11-7082 combined with varying concentrations of azithromycin or colistin for 24 h at 37°C before detection of bacterial growth at OD_600_ (blue line). **(A)** In combination with 1.8 μM 5-fluorouracil the IC_50_ of azithromycin decreased ~6-fold (6.4–1.1 μM). **(B)** In combination with 11.4 μM fluspirilene, the IC_50_ of colistin reduced ~30-fold (0.22 μM−7.4 nM). **(C)** In combination with 11.8 μM Bay 11-7082, the IC_50_ of colistin decreased ~4-fold (0.22 μM to 60 nM).

A drug combination of a standard care antibiotic agent with a newly identified compound from the above confirmed compounds (non-antimicrobial agents) was screened using the same AB5075 growth assay. After additional testing of these individual compounds with 25 standard care antibiotic therapies, three promising drug combination pairs with a synergistic effect against the drug resistant AB5075 strain were identified, including azithromycin/5-fluorouracil, colistin sulfate/fluspirilene, and colistin sulfate/Bay 11-7082. The synergistic effects of all three combination pairs were confirmed in a 96-well format assay. The IC_50_ values of known antibiotics was significantly reduced in the presence of the newly identified non-antibiotic agents. The IC_50_ value of azithromycin was reduced 6-fold, from 6.4 to 1.1 μM in the presence of 1.8 μM 5-fluorouracil (Figure 4A). Fluspirilene (11.4 μM) and Bay 11-7082 (11.8 μM) increased the inhibitory activity of colistin sulfate against the drug resistant AB5075 by 30- and 4-fold, respectively. The IC_50_ values of colistin were reduced from 0.22 to 0.0074 and 0.06 μM in the presence of fluspirilene and Bay 11-7082, respectively (Figures 4B,C).

## Discussion

In this study, we describe the optimization and validation of a high throughput growth assay to measure the viability of a multidrug resistant *Acinetobacter baumannii* strain, AB5075. The 1,536-well format of the bacterial growth assay enabled the quick screening of thousands of compounds and drug combination sets with low reagent costs. The results from 25 known antibiotics revealed that AB5075 is resistant to most β-lactams, aminoglycosides, quinolones, and macrolides. The data agreed with these reported by Jacobs et al. (2014).

Seven non-antimicrobial agents, either approved drugs or in clinical trials, were identified in the compound screening campaign; these were confirmed as novel inhibitors of AB5075. For consideration of potential clinical applications of the newly identified antimicrobial activity of these compounds, the human plasma drug concentration levels should be higher than their IC_90_ values or minimum inhibitory concentrations (MIC). Two drugs, among these seven confirmed antimicrobial agents, 5-fluorouracil and 6-thioguanine, met this criterion; these two drugs are potent anticancer medications. Five-fluorouracil is a fluoropyrimidine and is a broad-spectrum anticancer agent, typically used as a first line chemotherapy agent for colorectal cancer (Longley et al., 2003). The standard treatment dose of 5-fluorouracil for cancer patients is 370 mg/m^2^ daily with a reported C_max_ of 48.41 μg/mL (Bocci et al., 2000) which is higher than the IC_90_ (25.22 μg/mL) we found in the AB5075 growth inhibition assay. It has dual inhibition mechanisms including functioning as an alternative substrate resulting in miscoding DNA and RNA and inhibiting thymidylate synthase. The broad spectrum antimicrobial activity of 5-fluorouracil was observed as early as 1985 (Bodet et al., 1985). Anti-microbial activity of 5-fluorouracil has also been confirmed against *S. aureus* and *S. epidermidis* (Gieringer et al., 1986; Rangel-Vega et al., 2015). Mechanistic studies of antimicrobial activity are scant for 5-fluorouracil. However, the antimycotic mechanism of flucytosine suggests that 5-fluorouracil may share the same inhibitory mechanism against pathogens as it has against cancer (Vermes et al., 2000). Flucytosine, a prodrug, is converted into 5-fluorouracil following cell uptake.

Six-thioguanine is mainly used as a chemotherapy for myeloid leukemia and myeloid malignancies (Munshi et al., 2014). The recommended dose for acute non-lymphocytic leukemia patients is 2–3 mg/kg daily in an oral form as a single agent, or 75–200 mg/m^2^ daily when used as a combination therapy. At a dose of 65 mg/m^2^, the mean peak plasma concentration of 6-thioguanine ranges from 6–10 μM (Kovach et al., 1986). Although the plasma concentration of conventional dosage is lower than the IC_90_, it has been reported that 87 μM in human plasma has been achieved at a larger dose of 800–1200 mg/m^2^ (Presant et al., 1984; Kovach et al., 1986). As a guanosine structural analog, 6-thioguanine gets incorporated into DNA and RNA, blocking the biosynthesis of these two essential macromolecules. Additionally, 6-thioguanine hinders purine synthesis by inhibiting hypoxanthine phosphoribosyltransferase (Hprt). The bactericidal effect of 6-thioguanine against *S. aureus* and bacteriostatic effects against for *E. coli* and *S. typhimurium* were reported previously (Soo et al., 2016). Although there is no Hprt homolog in bacteria, the bacterial PRTases [Xanthine-guanine phosphoribosyltranferase (Gpt), hypoxanthine phosphoribosyltransferase (Hpt) and adenine phosphoribosyltransferase (Apt)], show substrate binding-site conservation with Hprt, suggesting that these bacterial PRTases could be the molecular targets of 6-thioguanine (Wensing et al., 2014).

We have noticed that the majority of non-antimicrobial drugs we found in this repurposing screen are anticancer drugs. Because most anticancer drugs are cytotoxic and have serious side effects, it is a reasonable concern for application of anticancer drugs to treat infectious diseases. However, repositioning of anticancer drugs for infectious diseases has been reported (reviewed in Soo et al., 2016) despite the concerns of potential side effects. For example, miltefosine has been approved to treat leishmaniasis in 2014 (Berman, 2015). The application of gallium compounds to control *P. aeruginosa* in patients with cystic fibrosis is completed its phase II clinical trial (ClinicalTrials.gov^1^). Five-fluorouracil has been implemented as a coating agent for central venous catheters to prevent the bacterial colonization during treatment (Walz et al., 2008). Therefore, treatment needs to balance the therapeutic benefit with potential side effects before considering use of the anticancer drugs for the severe infectious diseases.

Azithromycin has pronounced activity against *H. influenza* as well as most gram-negative bacteria including *Acinetobacter baumannii*. Recently, two important findings support the potential of anti-multidrug resistant *Acinetobacter baumannii* for azithromycin. Azithromycin exhibited better efficacy in cell culture media and animal models than the canonical bacterial culture media (Lin et al., 2015) and it was effective against lipopolysaccharide deficient colistin-resistant strains (García-Quintanilla et al., 2015). The AB5075 strain belongs to the latter case; in our study the minimal inhibitory concentration for 90% inhibition (MIC_90_) of azithromycin was 8.5 μg/mL, which is lower than the average from 15 clinical isolates of 64 μg/mL (Fernández Cuenca et al., 2003). However, using the breakpoint for *H. influenza*, 4 μg/mL from CLSI, AB5075 is not sensitive to azithromycin. The MIC_90_ value of azithromycin drops to a clinically attainable level through a synergistic effect with 5-fluorouracil in the two-drug combination therapy format.

The appearance of colistin resistance in *Acinetobacter baumannii* infections presents an urgent need for development of new therapeutics. Glycopeptides and hydrophobic compounds such as trimethoprim showed synergistic effects with colistin in an earlier study (Vidaillac et al., 2012). In this study, we identified two non-antimicrobial drugs which can resensitize the AB5075 strain to colistin, lowering the MIC_90_ of colistin to clinically achievable concentrations.

Fluspirilene belongs to the diphenylbutylpiperidine family, which were first-generation antipsychotics. The initial therapeutic dose is 2 mg weekly followed by 1–10 mg weekly injection for maintenance. The C_max_ of fluspirilene at 2 mg is about 200 pg/mL (Swart et al., 1998). Diphenylbutylpiperidines are dopamine D2 receptor antagonists and ameliorate the positive symptoms resulting from the hyperdopaminergic neurotransmission (Seeman, 1980). The finding of antifungal potential of fluspiriline supports a mechanistic study suggesting that fluspirilene likely inhibits the AB5075 growth by blockading the calcium-modulating protein, calmodulin (Butts et al., 2013). On the other hand, Bay 11-7082 is widely known as an IκB kinase (IKK) inhibitor. The molecular target of Bay 11-7082 remains unclear. From the chemical structural perspective, Bay 11-7082 is a phenyl vinyl sulfone related compound. The vinyl sulfone in conjugation with a nitrile group makes it a good Michael acceptor to interact with cysteine. The ability to inhibit cysteine proteases through an irreversible Michael addition were demonstrated in a recent study (Kerr et al., 2009). Notably, vinyl sulfone was identified as an anti-parasitic agent through this mechanism, suggesting cysteine proteases could be the molecular target of AB5075 inhibition (Kerr et al., 2009).

In conclusion, we have identified 43 approved drugs or drug candidates that significantly suppressed the growth of the multidrug resistant AB5075 strain including seven non-antimicrobial indication compounds. We also found three pairs of two drug combinations that exhibited synergistic effects with two known antibiotics against the AB5075 strain including azithromycin/5-fluorouracil, colistin sulfate/fluspirilene, and colistin sulfate/Bay 11-7082. These drug pairs are not contraindicated as only minor interactions between azithromycin and 5-fluorouracil have been reported which may be tolerable to patients. While these drug combination pairs may have the potential for clinical trials to treat multidrug resistant *Acinetobacter baumannii* infections, the other drugs found in this study may be useful for identification of new drugs scaffolds or new targets to combat this pathogen.

## Author Contributions

Y-SC, WS, and WZ conceived and designed the study. Y-SC, WS, and MX performed the experiments. MS performed the statistical analysis. MK and RS contributed materials. Y-SC wrote the first draft of the manuscript. All authors contributed to manuscript revision, read, and approved the submitted version.

### Conflict of Interest Statement

The authors declare that the research was conducted in the absence of any commercial or financial relationships that could be construed as a potential conflict of interest.

## Footnotes

^1^ClinicalTrials.gov. Available online at: https://clinicaltrials.gov/ct2/show/NCT02354859

## References

[B1] Berman J. (2015). Miltefosine, an FDA-approved drug for the ‘orphan disease’, leishmaniasis. Expert Opin. Orphan Drugs 3, 727–735. 10.1517/21678707.2015.1039510

[B2] Bocci G. Danesi R. Di Paolo A. D. Innocenti F. Allegrini G. Falcone A.. (2000). Comparative pharmacokinetic analysis of 5-fluorouracil and its major metabolite 5-fluoro-5,6-dihydrouracil after conventional and reduced test dose in cancer patients. Clin. Cancer Res. 6, 3032–3037. 10955781

[B3] Bodet C. A. Jorgensen J. H. Drutz D. J. (1985). Antibacterial activities of antineoplastic agents. Antimicrob. Agents Chemother. 28, 437–439. 10.1128/AAC.28.3.437 2416271 PMC180269

[B4] Butts A. DiDone L. Koselny K. Baxter B. K. Chabrier-Rosello Y. Wellington M.. (2013). A repurposing approach identifies off-patent drugs with fungicidal Cryptococcal activity, a common structural chemotype, and pharmacological properties relevant to the treatment of Cryptococcosis. Eukaryot Cell 12, 278–287. 10.1128/EC.00314-12 23243064 PMC3571299

[B5] Demain A. L. (1999). Pharmaceutically active secondary metabolites of microorganisms. Appl. Microbiol. Biotechnol. 52, 455–463. 10.1007/s002530051546 10570792

[B6] Deng M. Zhu M. H. Li J. J. Bi S. Sheng Z. K. Hu F. S.. (2014). Molecular epidemiology and mechanisms of tigecycline resistance in clinical isolates of *Acinetobacter baumannii* from a Chinese university hospital. Antimicrob. Agents Chemother. 58, 297–303. 10.1128/AAC.01727-13 24165187 PMC3910737

[B7] Fernández Cuenca F. Pascual A. Martinez Martinez L. Perea E. J. (2003). *In vitro* activity of azithromycin against clinical isolates of *Acinetobacter baumannii*. Rev. Esp. Quimioter. 16, 204–208. 12973458

[B8] Gallagher L. A. Ramage E. Weiss E. J. Radey M. Hayden H. S. Held K. G.. (2015). Resources for genetic and genomic analysis of emerging pathogen *Acinetobacter baumannii*. J. Bacteriol. 197, 2027–2035. 10.1128/JB.00131-15 25845845 PMC4438207

[B9] García-Quintanilla M. Carretero-Ledesma M. Moreno-Martinez P. Martin-Pena R. Pachon J. McConnell M. J. (2015). Lipopolysaccharide loss produces partial colistin dependence and collateral sensitivity to azithromycin, rifampicin, and vancomycin in *Acinetobacter baumannii*. Int. J. Antimicrob. Agents 46, 696–702. 10.1016/j.ijantimicag.2015.07.017 26391380

[B10] Gieringer J. H. Wenz A. F. Just H. M. Daschner F. D. (1986). Effect of 5-fluorouracil, mitoxantrone, methotrexate, and vincristine on the antibacterial activity of ceftriaxone, ceftazidime, cefotiam, piperacillin, and netilmicin. Chemotherapy 32, 418–424. 10.1159/000238445 3093153

[B11] Huang R. Southall N. Wang Y. Yasgar A. Shinn P. Jadhav A.. (2011). The NCGC pharmaceutical collection: a comprehensive resource of clinically approved drugs enabling repurposing and chemical genomics. Sci. Transl. Med. 3:80ps16. 10.1126/scitranslmed.3001862 21525397 PMC3098042

[B12] Inglese J. Auld D. S. Jadhav A. Johnson R. L. Simeonov A. Yasgar A.. (2006). Quantitative high-throughput screening: a titration-based approach that efficiently identifies biological activities in large chemical libraries. Proc. Natl. Acad. Sci. U.S.A. 103, 11473–11478. 10.1073/pnas.0604348103 16864780 PMC1518803

[B13] Jacobs A. C. Thompson M. G. Black C. C. Kessler J. L. Clark L. P. McQueary C. N.. (2014). AB5075, a highly virulent isolate of *Acinetobacter baumannii*, as a model strain for the evaluation of pathogenesis and antimicrobial treatments. MBio 5, e01076–e01014. 10.1128/mBio.01076-14 24865555 PMC4045072

[B14] Johansson B. (1992). A review of the pharmacokinetics and pharmacodynamics of disulfiram and its metabolites. Acta Psychiatr. Scand. Suppl. 369, 15–26. 10.1111/j.1600-0447.1992.tb03310.x 1471547

[B15] Kerr I. D. Lee J. H. Farady C. J. Marion R. Rickert M. Sajid M.. (2009). Vinyl sulfones as antiparasitic agents and a structural basis for drug design. J. Biol. Chem. 284, 25697–25703. 10.1074/jbc.M109.014340 19620707 PMC2757971

[B16] Kovach J. S. Rubin J. Creagan E. T. Schutt A. J. Kvols L. K. Svingen P. A.. (1986). Phase I trial of parenteral 6-thioguanine given on 5 consecutive days. Cancer Res. 46, 5959–5962. 3756933

[B17] Lin L. Nonejuie P. Munguia J. Hollands A. Olson J. Dam Q.. (2015). Azithromycin synergizes with cationic antimicrobial peptides to exert bactericidal and therapeutic activity against highly multidrug-resistant gram-negative bacterial pathogens. EBioMedicine 2, 690–698. 10.1016/j.ebiom.2015.05.021 26288841 PMC4534682

[B18] Longley D. B. Harkin D. P. Johnston P. G. (2003). 5-fluorouracil: mechanisms of action and clinical strategies. Nat. Rev. Cancer 3, 330–338. 10.1038/nrc1074 12724731

[B19] Marinelli F. (2009). Chapter 2. From microbial products to novel drugs that target a multitude of disease indications. Methods Enzymol. 458, 29–58. 10.1016/S0076-6879(09)04802-2 19374978

[B20] Munshi P. N. Lubin M. Bertino J. R. (2014). 6-thioguanine: a drug with unrealized potential for cancer therapy. Oncologist 19, 760–765. 10.1634/theoncologist.2014-0178 24928612 PMC4077447

[B21] Oikonomou O. Sarrou S. Papagiannitsis C. C. Georgiadou S. Mantzarlis K. Zakynthinos E.. (2015). Rapid dissemination of colistin and carbapenem resistant *Acinetobacter baumannii* in Central Greece: mechanisms of resistance, molecular identification and epidemiological data. BMC Infect. Dis. 15:559. 10.1186/s12879-015-1297-x 26653099 PMC4675053

[B22] Presant C. A. Denes A. E. Liu C. Bartolucci A. A. (1984). Prospective randomized reappraisal of 5-fluorouracil in metastatic colorectal carcinoma. A comparative trial with 6-thioguanine. Cancer 53, 2610–2614. 10.1002/1097-0142(19840615)53:12&lt;2610::AID-CNCR2820531207&gt;3.0.CO;2-9 6372981

[B23] Rangel-Vega A. Bernstein L. R. Mandujano-Tinoco E. A. García-Contreras S. J. García-Contreras R. (2015). Drug repurposing as an alternative for the treatment of recalcitrant bacterial infections. Front. Microbiol. 6:282. 10.3389/fmicb.2015.00282 25914685 PMC4391038

[B24] Seeman P. (1980). Brain dopamine receptors. Pharmacol. Rev. 32, 229–313. 6117090

[B25] Soo V. W. Kwan B. W. Quezada H. Castillo-Juarez I. Perez-Eretza B. Garcia-Contreras S. J.. (2016). Repurposing of anticancer drugs for the treatment of bacterial infections. Curr. Top. Med. Chem. 17, 1157–1176. 10.2174/1568026616666160930131737 27697046

[B26] Sun W. Weingarten R. A. Xu M. Southall N. Dai S. Shinn P.. (2016). Rapid antimicrobial susceptibility test for identification of new therapeutics and drug combinations against multidrug-resistant bacteria. Emerg. Microbes Infect. 5:e116. 10.1038/emi.2016.123 27826141 PMC5148025

[B27] Swart K. J. Sutherland F. C. van Essen G. H. Hundt H. K. Hundt A. F. (1998). Determination of fluspirilene in human plasma by liquid chromatography-tandem mass spectrometry with electrospray ionisation. J. Chromatogr. A 828, 219–227. 10.1016/S0021-9673(98)00635-9 9916308

[B28] Vermes A. Guchelaar H. J. Dankert J. (2000). Flucytosine: a review of its pharmacology, clinical indications, pharmacokinetics, toxicity, and drug interactions. J. Antimicrob. Chemother. 46, 171–179. 10.1093/jac/46.2.171 10933638

[B29] Vidaillac C. Benichou L. Duval R. E. (2012). *In vitro* synergy of colistin combinations against colistin-resistant *Acinetobacter baumannii*, Pseudomonas aeruginosa, and Klebsiella pneumoniae isolates. Antimicrob. Agents Chemother. 56, 4856–4861. 10.1128/AAC.05996-11 22751540 PMC3421898

[B30] Walz J. Luber J. Reyno J. Stanford G. Gitter R. Longtine K.. (2008). A multicenter randomized controlled clinical trial comparing central venous catheters impregnated with either 5-fluorouracil or chlorhexidine/silver sulfadiazine in preventing catheter colonization. Crit. Care 12(Suppl. 2):P40. 10.1186/cc626120711070

[B31] Wang Y. Jadhav A. Southal N. Huang R. Nguyen D. T. (2010). A grid algorithm for high throughput fitting of dose-response curve data. Curr. Chem. Genomics 4, 57–66. 10.2174/1875397301004010057 21331310 PMC3040458

[B32] Wensing A. Gernold M. Jock S. Jansen R. Geider K. (2014). Identification and genetics of 6-thioguanine secreted by Erwinia species and its interference with the growth of other bacteria. Mol. Genet. Genomics 289, 215–223. 10.1007/s00438-013-0805-1 24374865

[B33] Xie R. Zhang X. D. Zhao Q. Peng B. Zheng J. (2018). Analysis of global prevalence of antibiotic resistance in *Acinetobacter baumannii* infections disclosed a faster increase in OECD countries. Emerg. Microbes Infect. 7:31. 10.1038/s41426-018-0038-9 29535298 PMC5849731

[B34] Zheng W. Sun W. Simeonov A. (2018). Drug repurposing screens and synergistic drug-combinations for infectious diseases. Br. J. Pharmacol. 175, 181–191. 10.1111/bph.13895 28685814 PMC5758396

